# Supplementary material for: Genomic Analysis Revealed the International and Domestic Transmission of Carbapenem-Resistant Klebsiella pneumoniae in Chinese Pediatric Patients
Source: Microbiol Spectr. 2023 Mar 1;11(2):e03213-22. doi: 10.1128/spectrum.03213-22 (PMC10101082; doi:10.1128/spectrum.03213-22)
Supplement: Supplemental file 3 — Fig. S1 to S4. Download spectrum.03213-22-s0003.pdf, PDF file, 1.6 MB [file spectrum.03213-22-s0003.pdf]

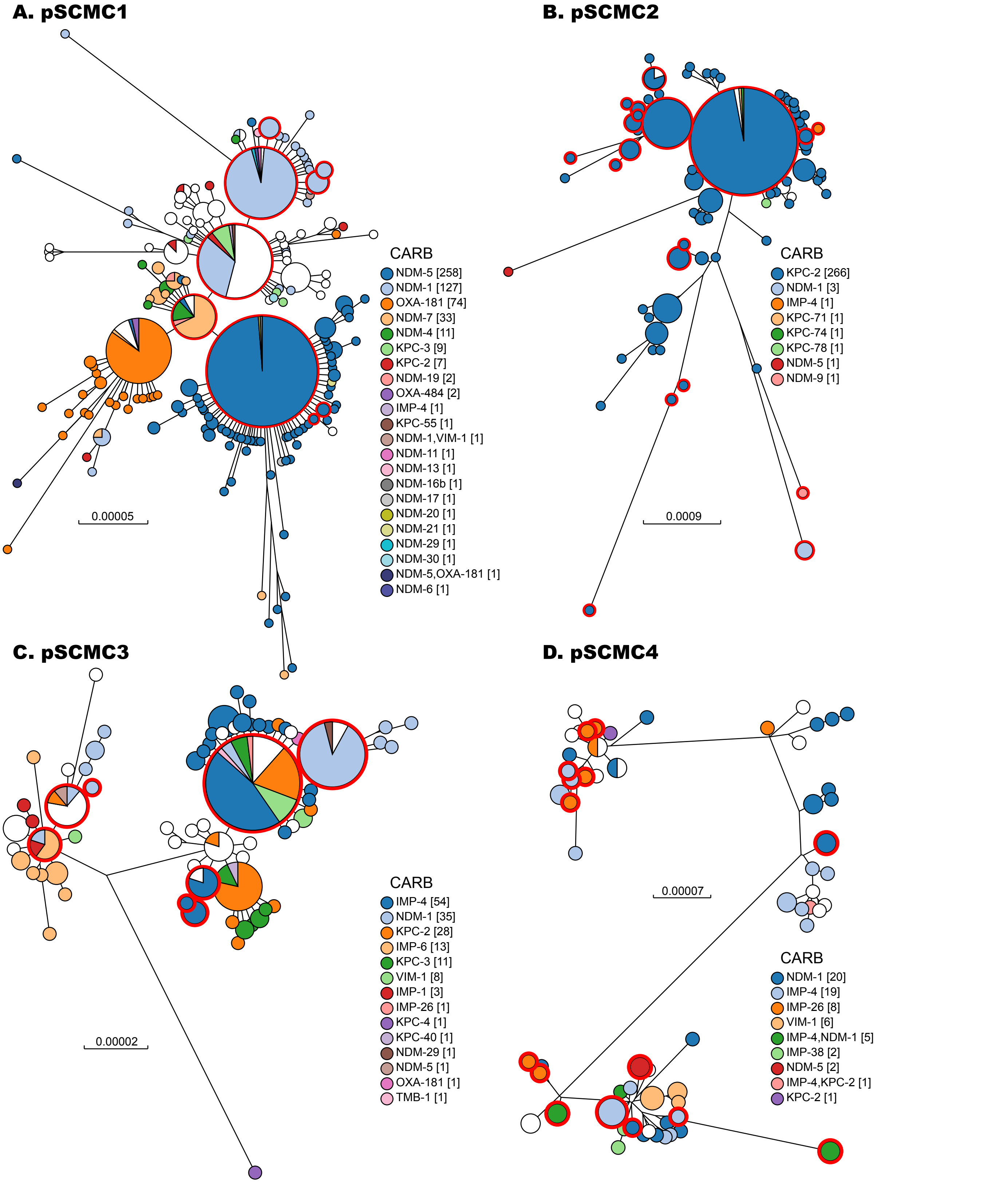

Supplementary Figure 1. Maximum-likelihood phylogeny of the four predominant plasmids including both Shanghai isolates and those from public databases. Isolates were color-coded according to different carbapenem resistance genes. The isolates from present study were marked as red circles. (A&C) The majority of pSCMC1 and pSCMC3 carried NDM genes. (B&D) The pSCMC2 and pSCMC4 were associated with KPC-2 and IMP gene. Abbreviations: SCMC, Shanghai Children's Medical Center; NDM, New Delhi metallo-β-lactams; KPC-2, *Klebsiella pneumoniae* carbapenemase; IMP, Imipenemase.

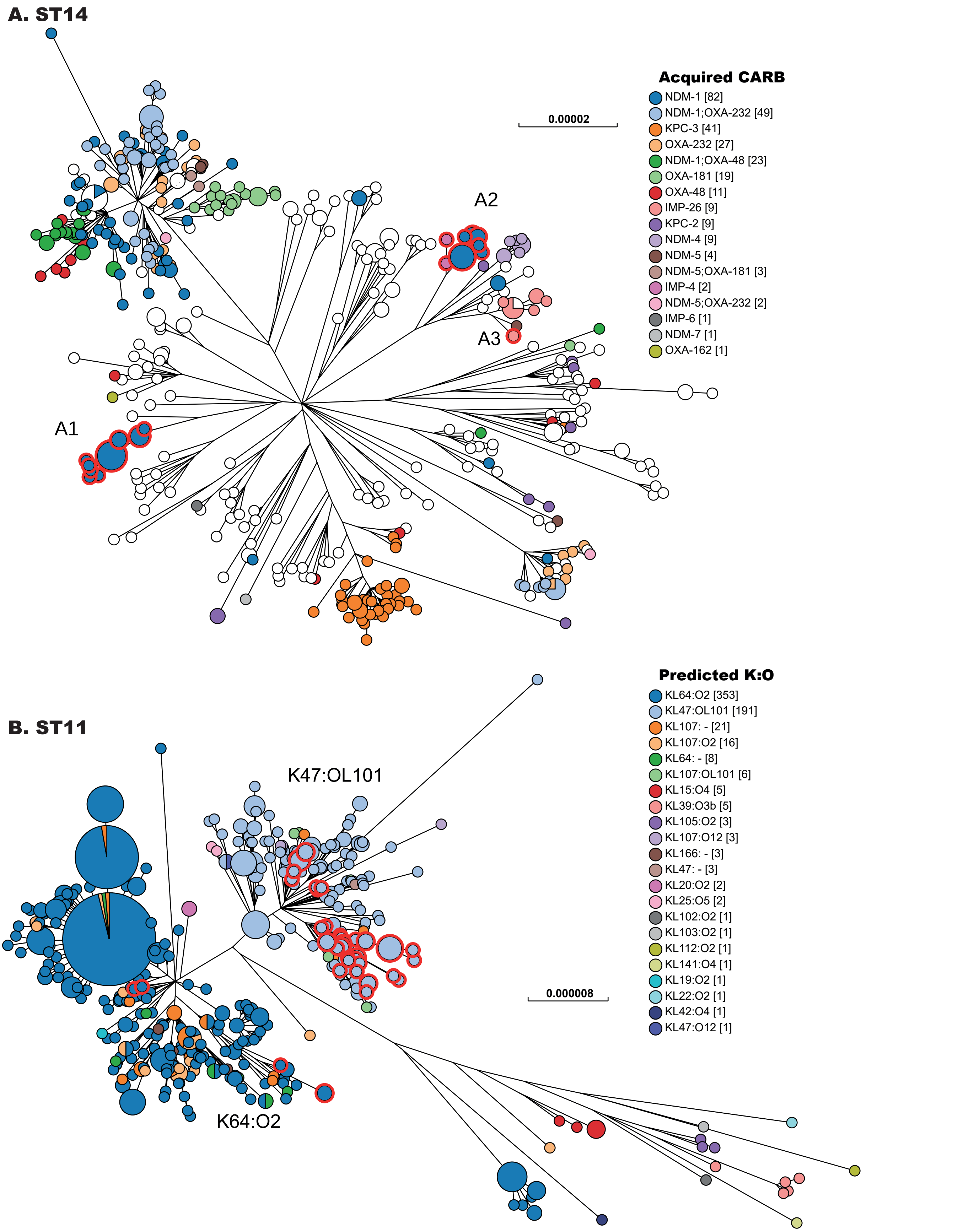

Supplementary Figure 2. (A) Maximum-likelihood phylogeny of *K. pneumoniae* ST14 and ST11 isolates in global scale. (A) The ST14 isolates were color-coded according to their carried carbapenem resistance genes. The ST14-NDM-1 CRKP isolated from this study in Shanghai were clustered into two major clades. (B) Phylogeny of all Chinese ST11 genomes. The ST11 genomes were color-coded according to their K:O type. The red circles indicated SCMC isolates, which were separated into two clades of ST11-KL64 and ST11-KL47. Abbreviations: ST: sequence types; SCMC, Shanghai Children' s Medical Center; NDM, New Delhi metallo-β-lactams; CRKP, carbap enem-resistant *K. pneumoniae*; IMP, Imipenemase.

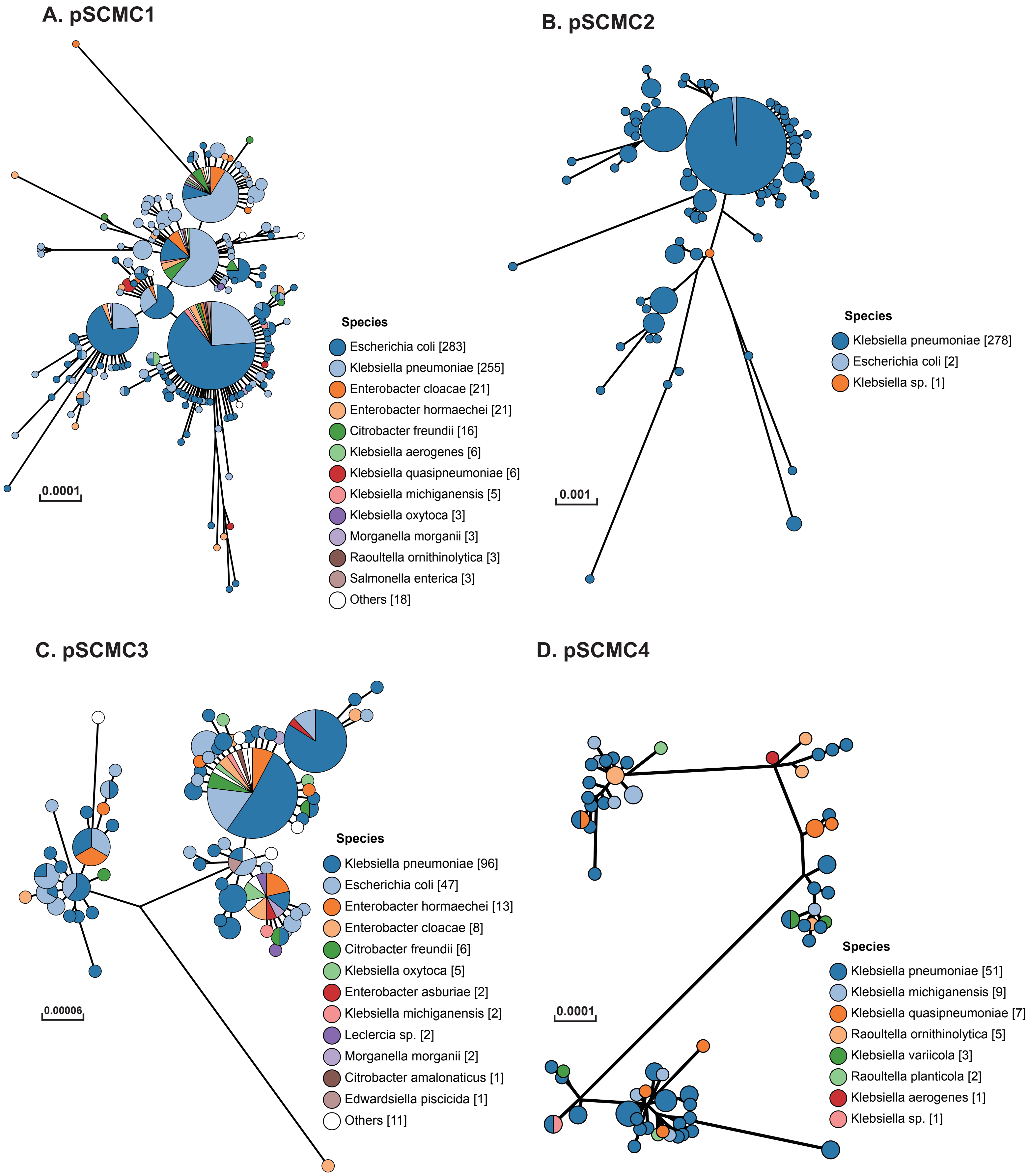

Supplementary figure 3. Maximum-likelihood phylogeny of the four predominant plasmids including both Shanghai isolates and those from public databases. Isolates were color-coded according to bacterial species. pSCMC 1 and pSCMC 3 (A&C) were carried by >20 *Enterobacteriaceae* species, while mostly pSCMC 2 and pSCMC 4 (B&D) were restricted to *K.pneumoniae*. Abbreviations: SCMC, Shanghai Children's Medical Center.

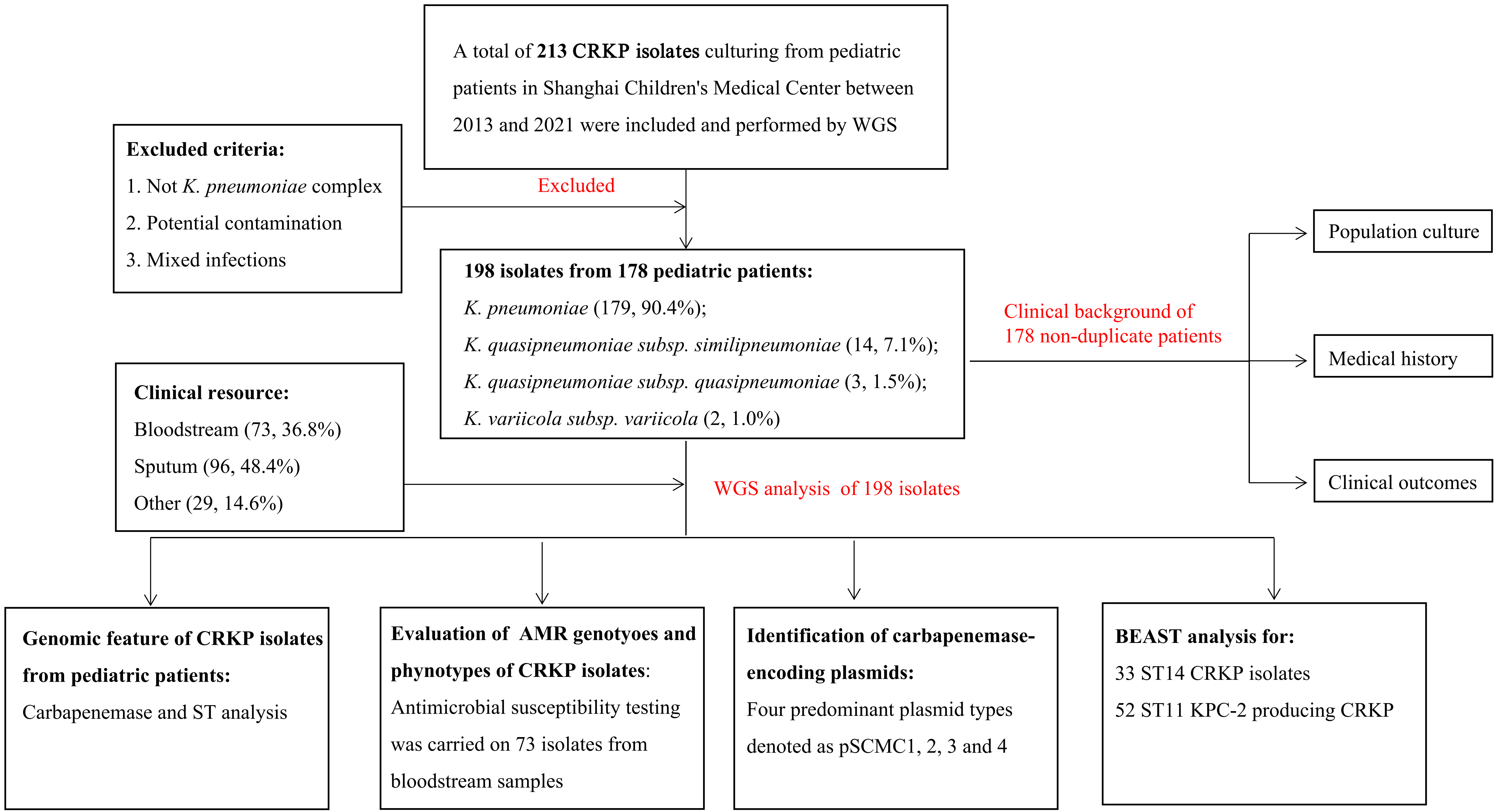

Supplementary Figure 4. Flow chart diagram of methods for sampling and charactering of carbapenem-resistant *Klebsiella pneumoniae* in pediatric patients from Shanghai Children's Medical Center. Abbreviations: CRKP, Carbapenem-resistant *Klebsiella pneumoniae*; WGS, Whole-genome sequencing; AMR, Antimicrobial resistance; ST, Sequence types; NDM, New Delhi metallo-β-lactams; KPC-2, *Klebsiella pneumoniae* carbapenemase.
